# Development of *3D-iNET ORION*: a novel, pre-clinical, three-dimensional in vitro cell model for modeling human metastatic neuroendocrine tumor of the pancreas

**DOI:** 10.1007/s13577-024-01113-7

**Published:** 2024-08-05

**Authors:** Jan Strnadel, Mark A. Valasek, Grace Y. Lin, Huahui Lin, Ann M. Ponsford Tipps, Sang Myung Woo, Ken Fujimura, Huawei Wang, Sunkyu Choi, Jack Bui, Christopher Hermosillo, Kristen Jepsen, Michael R. Navarro, Jonathan A. Kelber, Richard L. Klemke, Michael Bouvet

**Affiliations:** 1 Jessenius Faculty of Medicine in Martin, Biomedical Centre Martin, Comenius University in Bratislava, 036 01 Martin, Slovakia; 2 Department of Pathology, University of California, La Jolla, San Diego, CA USA; 3 Research Institute, Center for Liver and Pancreatobiliary Cancer, National Cancer Center, Goyang-si, Republic of Korea; 4 Proteomics Core, Weill Cornell Medicine-Qatar, Qatar Foundation, Education City, Doha, Qatar; 5 Moores Cancer Center, University of California, La Jolla, San Diego, CA USA; 6 Institute for Genomic Medicine, University of California, La Jolla, San Diego, CA USA; 7 Neuroregeneration Laboratory, Department of Anesthesiology, University of California San Diego, La Jolla, San Diego, CA USA; 8 Department of Biology, Baylor University, Waco, TX USA; 9 Division of Surgical Oncology, Department of Surgery, Moores Cancer Center, University of California San Diego, La Jolla, San Diego, CA USA

**Keywords:** Neuroendocrine tumor, Pancreas, 3D cancer cell lines

## Abstract

Neuroendocrine tumors (NETs) of the pancreas are rare neoplasms that present complex challenges to diagnosis and treatment due to their indolent course. The incidence of pancreatic neuroendocrine tumors has increased significantly over the past two decades. A limited number of pancreatic neuroendocrine cell lines are currently available for the research. Here, we present 3D-iNET ORION, a novel 3-dimensional (spheroid) cell line, isolated from human pancreatic neuroendocrine tumor liver metastasis. Three-dimensionally grown (3D) cancer cell lines have gained interest over the past years as 3D cancer cell lines better recapitulate the in vivo structure of tumors, and are more suitable for in vitro and in vivo experiments. 3D-iNET ORION cancer cell line showed high potential to form tumorspheres when embedded in Matrigel matrix and expresses synaptophysin and EpCAM. Electron microscopy analysis of cancer cell line proved the presence of dense neurosecretory granules. When xenografted into athymic mice, 3D-iNET ORION cells produce slow-growing tumors, positive for chromogranin and synaptophysin. Human Core Exome Panel Analysis has shown that 3DiNET ORION cell line retains the genetic aberration profile detected in the original tumor. In conclusion, our newly developed neuroendocrine cancer cell line can be considered as a new research tool for in vitro and in vivo experiments.

**Supplementary Information:**

The online version contains supplementary material available at 10.1007/s13577-024-01113-7.

## Background

Pancreatic neuroendocrine tumors represent a heterogeneous group of tumors that can be divided into well-differentiated endocrine tumors, well-differentiated endocrine carcinomas, and poorly differentiated endocrine carcinomas [1, 2]. Although rare (only about 1–3% of all pancreatic neoplasms are neuroendocrine tumors), these tumors pose a big challenge for treatment mainly because of their resistance to systemic therapy. The incidence of pancreatic NET increased significantly over the past two decades. Neuroendocrine tumors appear at all ages, with the highest incidence being reported from the fifth decade onward. Most (~ 85%) of the pancreatic neuroendocrine tumors are functional and produce unique clinical and metabolic characteristics [3]. Based on the hormone secreted, they are classified as insulinomas, gastrinomas, glucagonomas, vasoactive intestinal peptide secreting tumors (VIPomas) and somatostatinomas [3, 4]. Although patients with neuroendocrine tumors have relatively good prognosis (when compared with other types of cancer), these tumors frequently metastasize to the liver [5]*.* The presence of liver metastasis is a negative prognostic factor for long-term survival of patients [6].

Here we describe the establishment and characterization of a new, three- dimensional neuroendocrine cancer cell line obtained from pancreatic neuroendocrine tumor liver metastasis and grown in the form of 3D spheroids from the time of isolation. Cancer cells cultured in 3D environment more closely resemble in vivo tissue architecture when compared to monolayer (2D) culture. 3D culture methods, although known for years, are being adopted more recently thanks to availability of novel, bioengineered substrates. Multiple variations of 3D culturing methods exist—cells are cultured as aggregates, on 3D scaffolds or embedded in gels [7]. In this study, 3D protein matrix that mimics and reassembles complex natural tumor environment was used for establishing and maintenance of the 3D-iNET ORION line.

## Materials and methods

### Clinical case and tumor biopsy isolation

**Fig. 1:**
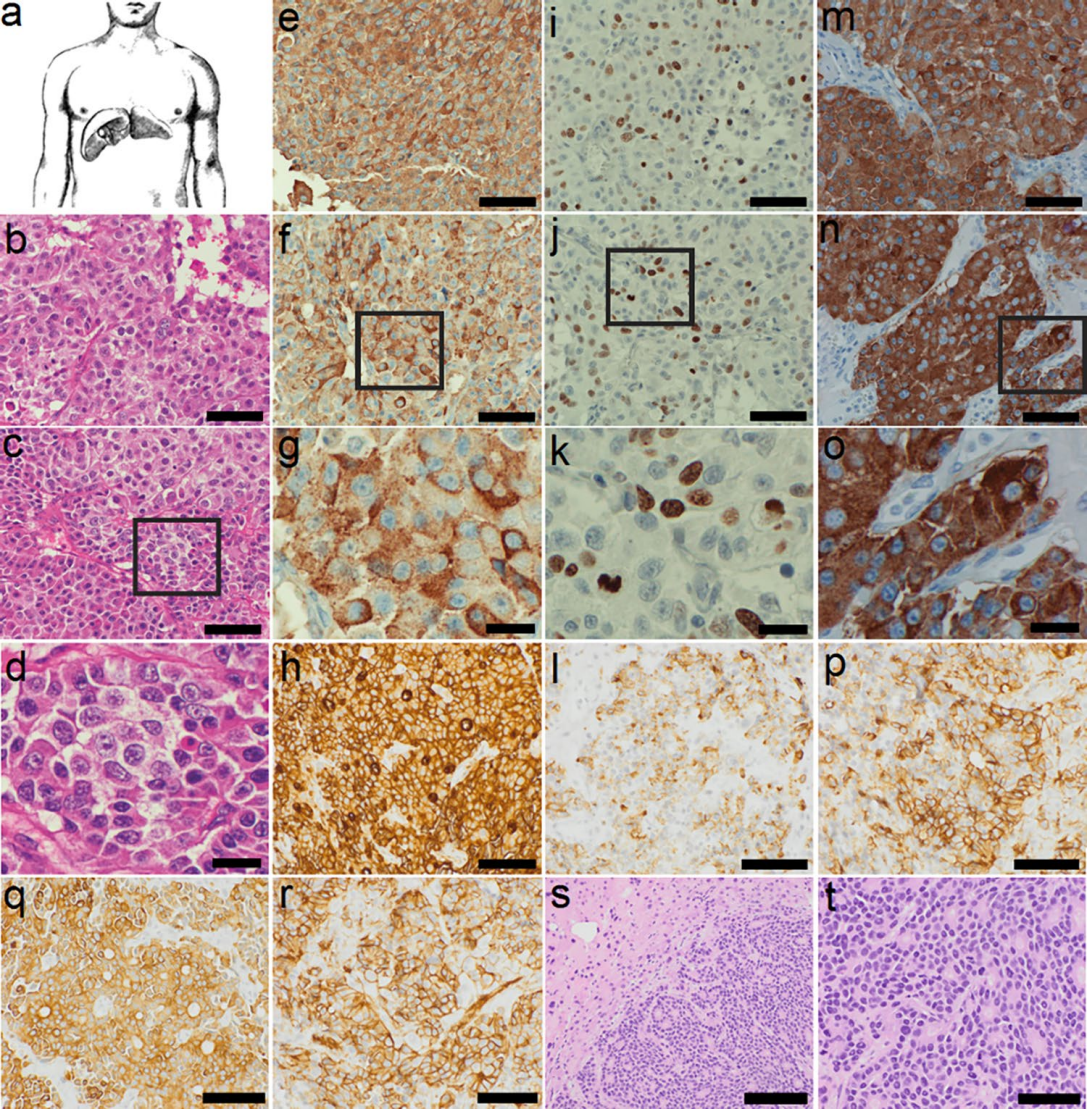
*3DiNET ORION* cell line was derived from a 83-year-old male patient with pancreatic neuroendocrine tumor liver metastasis (**a**). Histological evaluation of tumor shows the infiltration of liver parenchyma by sheets of small, uniform tumor cells focally arranged in rosettes (**b**–**d**). The tumor cells showed strong positivity for chromogranin (**e**–**g**) and synaptophysin (**m**–**o**). NET tumor was also positive for CEA (**h**), CD99 (**p**), CK7 (**q**), and CK19 (**r**). Ki-67 marker was found positive in approximately 70% of the tumor cells, consistent with high proliferation rate (**i**–**k**). Patchy positivity was detected for CD56 (**l**). Ki-67 marker was found positive in approximately 70% of the tumor cells, consistent with a high proliferation rate (**i**–**k**), although mitotic figures were not prominent. Images were captured at 4 × , 10 × and 20 × magnification. Scale bars represent 50 µm (**b**, **c**, **e**, **f** and **i**), 20 µm (**d**, **g**, **k** and **o**) and 100 µm (**h**, **l** and **p**)

Tumor tissue was extracted by laparoscopic liver biopsy (Fig. 1a) from an 83-year-old male patient with history of pancreatic cancer, at UCSD Medical Center, San Diego, USA. The specimen showed the infiltration of liver parenchyma by sheets of small, uniform tumor cells focally arranged in rosettes. Lymphovascular invasion was identified in the sample and some of the rosettes showed luminal blue mucin (Fig. 1b–d and Fig. 1t). Rare cells also showed intracytoplasmic mucin (Fig. 1b–d, t).

The tumor cells showed strong positivity for chromogranin (Fig. 1e–g) and synaptophysin (Fig. 1m–o), CEA (Fig. 1h), CD99 (Fig. 1p), CK7 (Fig. 1q), and CK19 (Fig. 1r). Patchy positivity was detected for CD56 (Fig. 1l). Ki-67 marker was found positive in approximately 70% of the tumor cells, consistent with a high proliferation rate (Fig. 1i–k), although mitotic figures were not prominent. Overall, the immunohistologic features were most consistent with grade 3 pancreatic neuroendocrine tumor (G3 Pan NET). Before surgery, patient signed informed consent letter, and donated part of the tissue for research project, previously approved by Institutional Review Board committee (IRB # 071136X).

## Isolation of cells from tumor biopsy

Small piece of the tumor tissue was obtained after surgical removal of tumor (UCSD Thornton Hospital, La Jolla, San Diego) and transferred to the lab on ice in sterile cell culture medium (DMEM/F12 Glutamax, supplemented with 10% FBS and 1% P/S, (Gibco)). Tissue was washed 3 times in BSL-2 class laminar hood with sterile PBS supplemented with antibiotic/antimycotic (Gibco) and minced into 1–3 mm pieces with a sterile surgical blade. To obtain single-cell suspension, pieces of tumor were mixed with collagenase (Type IA,  ≥ 125 CDU/mg, Sigma-Aldrich), reconstituted with HBSS, and incubated at 37 °C for 45 min. Solution with digested tissue was then filtered through 40 µm nylon mesh strainer (Falcon). Cells were washed 3 × with HBSS and plated on 6-well ultra-low attachment plate (corning) in media containing DMEM/F12, bFGF, EGF, B27, KOSR, ROCK inhibitor, and EDTA (full protocol is described in reference). Culture dish was checked periodically (every day) for appearance of spheroids. Once spheroids were visible (in around 3 to 4 weeks after plating), they were collected, washed with PBS and embedded in 1:1 mixture of media and matrigel (corning) extracellular matrix protein mixture. One part of the tumor tissue was processed as follows: explant culture was used to obtain population of stromal cells or cancer-associated fibroblasts. Briefly, small pieces of NET tumor tissue were placed on the bottom of cell culture plastic dish and cultured for few days in medium, containing DMEM/F12 glutamax, supplemented with 10% FBS and 1% P/S (gibco). Stromal cells that started to appear on the border of tumor tissue 3–6 days later were expanded to full confluency and used as “feeder layer” for small spheroids, cultured above the feeder cells. The list of antibodies used for IHC is in Supplementary Table 1.

### Cryopreservation of cells or spheroids

Cells and spheroids were cryopreserved in DMEM/F12 Glutamax culture medium, containing 10% DMSO (Sigma Aldrich), with Mr.Frosty (Nalgene, USA) freezing container. Vials with frozen spheroids/cells were stored in vapor phase of liquid nitrogen.

## STR analysis

The cell line identity and originality was tested with human cell authentication service using FTA sample collection kit. Briefly, cells isolated from primary tumor and cells from established 3D-iNET ORION cell line were prepared in laminar hood according to manufacturer´s recommendation and sent for analysis to ATCC (American type culture collection, Manassas, Virginia, USA).

### FACS analysis

Spheres isolated from Matrigel were rinsed with sterile phosphate-buffered saline and incubated with accutase at 37 °C for 45 min. A single-cell suspension was obtained by filtering through a 40 µm cell strainer (BD Biosciences, USA). Cells were rinsed twice with sterile phosphate-buffered saline and cell viability was assessed by Trypan blue exclusion staining and counting with the Countess Cell Counter (Invitrogen, USA). Blocking step was performed in PBS with 1% mouse serum for 30 min at 4 °C. Cells were then incubated with primary antibodies directly conjugated with fluorochromes for 45 min on ice. Blocking solution supplemented with EDTA (0.05%) was used for dilution of primary antibodies. Cells were then washed twice with PBS and re-suspended in 0.3 mL PBS with 1% mouse serum and 0,05% EDTA, and analyzed by FACS Canto II flow cytometer (Becton–Dickinson, USA). Gates and dot-blots were processed with FlowJo software. The list of antibodies used for flow cytometry analysis is in Supplementary Table 1.

### Western blot analysis

Cell extracts for western blot analysis were harvested directly from spheres with RIPA buffer (Cell Signaling, USA) freshly supplemented with protease and phosphatase inhibitors (Roche, Germany). Protein concentration was normalized for each sample with BCA Protein assay (Thermo-scientific, USA). Immunoblotting was performed using nitrocellulose membranes and 4–12% gradient SDS polyacrylamide gels (Invitrogen, USA). Membranes were blocked in 5% nonfat milk in Tris-buffered saline with Tween-20 (TBST, pH 7.4). Primary antibodies (diluted in blocking buffer) were incubated with membranes overnight at 4 ºC. Membranes were washed three times for 10 min on shaker in washing buffer (TBST, pH 7.4 containing 0.05% Tween 20. Secondary antibodies were diluted in blocking buffer and incubated for 1 h at laboratory temperature. After washing, membranes were incubated with WestPico chemiluminescent solution (Pierce, USA) and signal was detected using Kodak BlueFilm. Subsequently, films were scanned using standard office scanner. The list of antibodies used for Western blot protein analysis is in Supplementary Table 1.

### Antibody array

Protein lysates were isolated with lysis buffer provided with the kit. Membranes with immobilized array of antibodies were processed with samples according to manufacturer`s recommendation. Chemiluminescent signal was detected with KODAK Blue Film and films were scanned with scanner.

### IHC staining

Staining was performed on 4-micron paraffin tissue sections placed on Superfrost Plus (Fisher Scientific, USA) slides. Sections were deparaffinized and cleared before being treated for endogenous peroxidase activity in 3% solution of hydrogen peroxide (H_2_O_2_) and methanol. This treatment was followed by hydration in a graded alcohol series to distilled water. Subsequently, sections were microwave-treated for 12 min in citric acid buffer. Slides were allowed to cool and placed in PBS before adding of antibodies. Sections were incubated with 10% universal blocker (Biogenex, USA) for 20 min. in a humidity chamber. Slides were blotted and covered with primary antibody in a humidity chamber and incubated overnight at room temperature. After washing with PBS, slides were incubated for 60 min with biotinylated universal link antibody (Biogenex, USA). Two 5 min rinses in PBS and 30 min incubation with streptavidin–peroxidase complex completed the staining. Color development was achieved by treatment with the chromogen DAB (Vector Laboratories, Redwood City, USA) and was carried out for 3–5 min under a microscope. The slides were rinsed in running tap water, counter-stained in hematoxylin, dehydrated, cleared and covered. Negative control slides were run without primary antibody. The list of antibodies used for IHC is in Supplementary Table 1.

### Fluorescent and bright field microscopy

Phase contrast and fluorescent imaging was performed using a Nikon Eclipse Ti inverted fluorescent microscope running Nikon Elements software and equipped with a temperature-controlled chamber and Hamamatsu Orca CCD camera. The list of antibodies used for fluorescent microscopy is in Supplementary Table 1.

### *Electron* microscopy

Spheroids isolated from Matrigel (Corning) with dispase enzyme digestion (as described in Material and Methods section) were immersed in modified Karnovsky’s fixative (2.5% glutaraldehyde and 2% paraformaldehyde in 0.15 M sodium cacodylate buffer, pH 7.4) for at least 4 h, post-fixed in 1% osmium tetroxide in 0.15 M cacodylate buffer for 1 h and stained *en bloc* in 2% uranyl acetate for 1 h. Samples were dehydrated in ethanol, embedded in Durcupan epoxy resin (Sigma-Aldrich), sectioned at 50–60 nm on a Leica UCT ultramicrotome, and picked up on Formvar and carbon-coated copper grids. Sections were stained with 2% uranyl acetate for 5 min and Sato’s lead stain for 1 min. Grids were viewed using a JEOL 1200EX II (JEOL, Peabody, MA) transmission electron microscope and photographed using a Gatan digital camera (Gatan, Pleasanton), or viewed using a Tecnai G^2^ Spirit BioTWIN transmission electron microscope equipped with an Eagle 4 k HS digital camera (FEI, Hilsboro).

### Xenografting of spheroids into nude mice

When spheroids reached the size around 100–200 µm, they were isolated from Matrigel matrix by treatment with dispase (Stem Cell Technologies), for 30 min, (at 37 °C), washed with PBS with 0.005 M EDTA (to stop the reaction) and twice with PBS only. Individual spheres were mixed with 10% GFR Matrigel in sterile PBS and directly implanted subcutaneously into 6-week-old female Crl:NU(NCr)-Foxn1nu (athymic) mice (The Jackson Laboratory). Total of 3 mice were used for grafting experiment. The animals were monitored weekly for growth of tumors. When the tumor xenografts reached around 15 mm in size (approximately 5–6 months after grafting), animals were sacrificed by CO_2_ inhalation and tumors isolated for fixation and further analysis. Small piece of sample was placed in 10% formalin for paraffin embedding and for H&E staining. Animals were maintained in accordance with the standards of the University of California, San Diego.

## Specimen fixation

Samples were fixed in 10% buffered formalin for 72 h and embedded in paraffin. For histopathological analysis, tissue was serially sectioned (3 mm thick) and every ten sections stained with hematoxylin and eosin (H&E). Remaining sections were kept for immunohistochemical studies. Following incubation with the primary antibodies, positive cells were visualized using 3,3-diaminobenzidine tetrahydrochloride plus (DAB +) as a chromogen.

## Human core exome panel analysis

Genomic analysis and identity testing was performed using the Human Core Exome array. Three samples were analyzed in quadruplicates (primary tumor, 3D-iNET ORION cells (passage no.13) and 3D-iNET ORION cells (passage no.20). Briefly, total of 200 ng of DNA (isolated with Qiagen Blood and Tissue DNeasy kit) was hybridized to Infinium HumanCoreExome-24 arrays (Illumina), and stained per Illumina’s standard protocol. Copy Number Variation (CNV) calling was carried out in Nexus CN (version 7.5) and manually inspected, visualizing the B-allele frequencies (proportion of A and B alleles at each genotype) and log R ratios (ratio of observed to expected intensities) for each sample, as described previously [8].

## Results

### Isolation of cell line

**Fig. 2:**
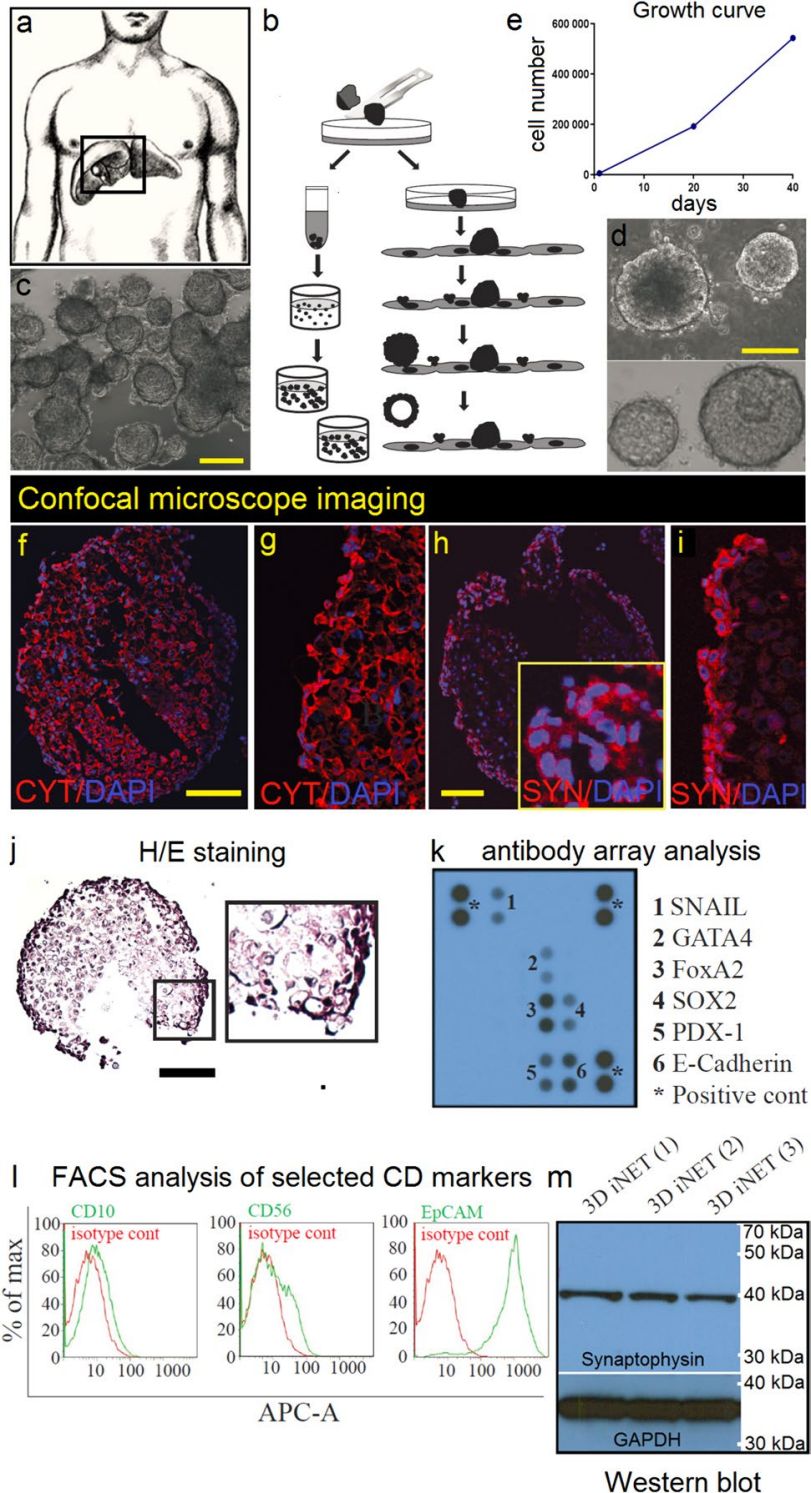
Primary 3D spheroid cell line was derived with two different strategies—metastatic tumor tissue (**a**) was processed as described previously [23] and also with modified protocol that utilizes cancer-associated fibroblasts or stromal cells, isolated from the same tumor, as a “feeder layer” (see *Material and Methods* section for detailed protocol description). A continuously growing cell line (**c** and **d**, scale bar represents 500 µm) was finally isolated with the use of the first approach (**b** left part of the scheme) utilizing Matrigel-embedded spheroids [22]. For growth curve construction and doubling time analysis, cells growing in the form of spheroids (at passage No.22) were used (**e**). Confocal microscope imaging of cryosectioned spheres showed strong positivity of *3DiNET ORION* cells for cytokeratins (**f**, **g**) and synaptophysin (**h**, **i**). Image **j** shows hematoxylin–eosin staining of cryosectioned sphere (bar represents 500 µm). Expression of several transcription factors expressed by *3DiNET ORION* cells was detected by antibody array protein chip (**k**). Expression of selected CD markers (CD10, CD56 and EpCAM) was evaluated by flow cytometry (**l**), while expression of synaptophysin was verified in lysates from three independent experiments (cells from passage No.7 (1st line), No.13 (2nd line) and No.20 (3rd line) was used) by Western blot analysis (GAPDH served as loading control), **m**)

3D spheroid cell line was successfully isolated and propagated in 3D culture in Matrigel matrix and expanded for almost 21 passages for over two years. Although some signs of cell proliferation were visible in these plates, due to the limited number of cancer cells and “easy-to-peel” character of feeder layer, this approach was eventually stopped and culturing the 3D-iNET ORION spheroids continued only in 3D culture with Matrigel. During this period, spheroids did not show any signs of phenotypical changes or changes in growth dynamic (Fig. 2a–e).

### Characterization of cell line

Doubling time of 3D-iNET ORION spheroid cell line was evaluated based on counting the number of cells isolated at various time-points (day 0, day 20th and 40th day, Fig. 2e) from spheroids and calculated to be around 6 days. Cells from spheroids from earlier passage (P7) were subjected to ICC, FACS and Western blot analysis and protein microarray analyses (Fig. 2j–l). Confocal imaging showed expression of cytokeratins and synaptophysin in spheroids formed by 3D-iNET ORION cells (Fig. 2f–i). Synaptophysin expression was verified in 3 independent samples by Western blot (Fig. 2m). Expression of cell surface markers was evaluated with flow cytometry on single cells prepared with accutase digestion of spheroids (Fig. 2l). Expression of CD10 (common acute lymphoblastic leukemia antigen (CALLA)) was detected in about ~ 4% of the cells. CD56 (neural cell adhesion molecule, or NCAM) was detected in about 8.4% of total cells. EpCAM (epithelial cell adhesion molecule) was highly expressed in 94.7% of cells, isolated from spheroids (Fig. 2l). To investigate the expression of intracellular transcription factors, protein array analysis of 3D-iNET ORION spheroids lysates was performed (Fig. 2k). Protein array analysis showed expression of multiple intracellular transcription factors involved in neuroendocrine cancer pathobiology—Snail, GATA4, FoxA2, Sox-2, PDX-1 and surface molecule (E-cadherin).

### *Electron* microscopy evaluation

**Fig.3:**
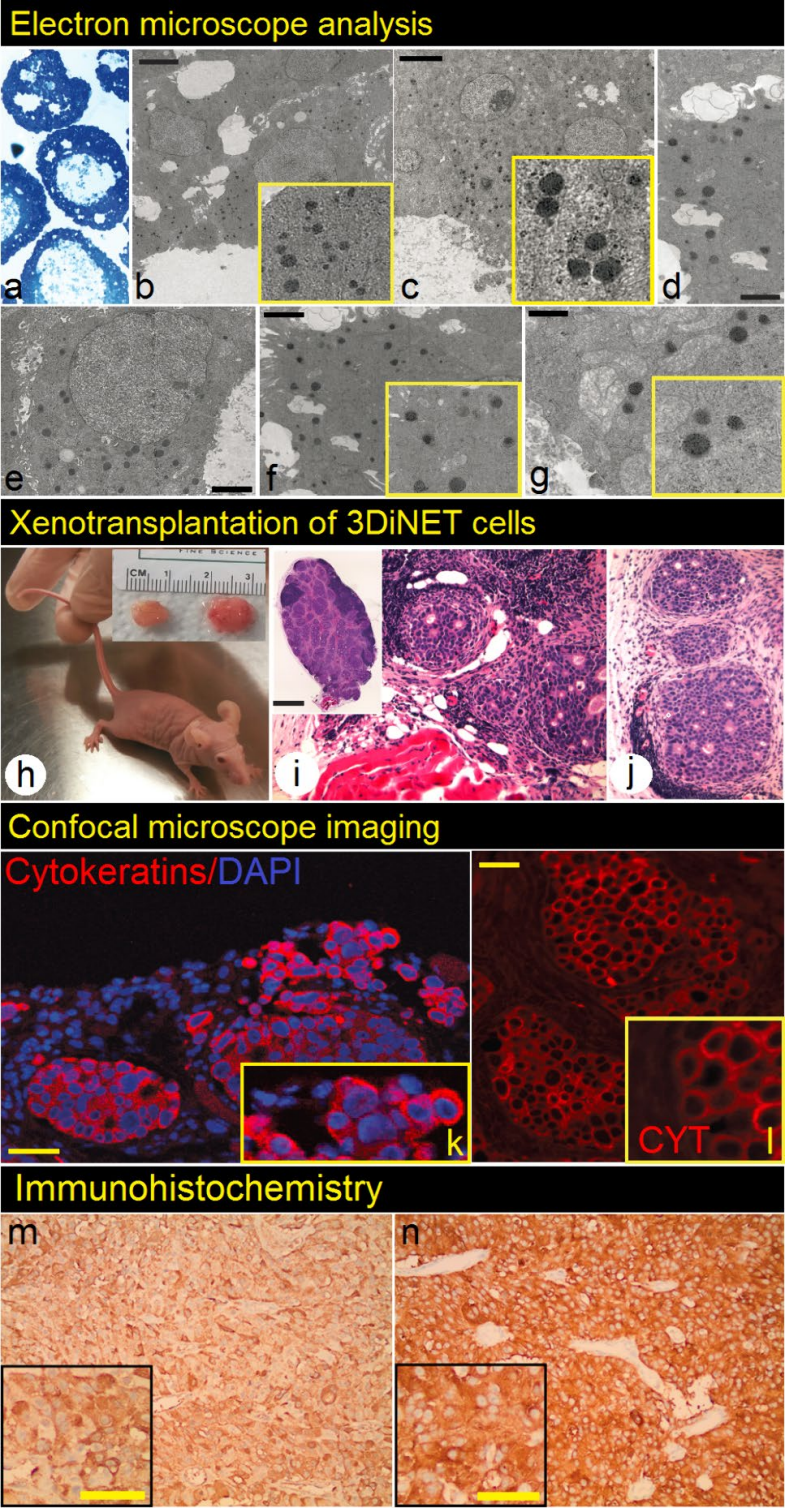
Characterization of *3DiNET ORION* cell line at in vitro and in vivo level. Ultrathin (50–60 nm), resin-embedded sections (**a**) prepared from 3D spheroids and stained with uranyl acetate and Sato´s lead stain, were analyzed using transmission electron microscope. The presence of dense neurosecretory granules (evidence for neuroendocrine origin of the tumor) was detected (**b**–**g**).Scale bars 1000 nm. represent Subcutaneous bilateral xenotransplantation of *3DiNET ORION* cell line in the form of spheroids resulted into the formation of tumors, approximately 3 months after xenografting into athymic (nude) mice (**h**, **i** and **j** shows H/E staining of tumors). Confocal imaging of cryosections (**k**, **l**) prepared from xenografts showed strong positivity for cytokeratins. **m** shows positivity for chromogranin and **n** for synaptophysin Scale bars represent 50 µm

Electron microscope imaging of ultra-thin plastic sections prepared from spheroids was used for detection of neuroendocrine granules. These subcellular structures with typical morphology (Fig. 3a–g) provide evidence for neuroendocrine origin of the tumor. 3D-iNET ORION showed clear presence of neurosecretory granules with various densities (Fig. 3a–g).

## STR analysis

STR analyses of primary tumor and 3D-iNET ORION cancer cell line (passage no.20) have shown that both samples were human origin and were derived from the same patient (Supplementary Fig. 1). STR analysis of 3D-iNET ORION cell line proved that cell line is original and does not match any cell line profile in ATCC STR database.

### Tumorigenicity of the cell line and xenografting

Spheroids that were isolated from 3D matrix with dispase treatment, engrafted successfully in 6-week-old female nude mice [*n* = 3, bilateral injection]. Palpable tumors appeared in ~ 3 months after grafting and progressed slowly during the following 3 months. Animals grafted with the 3D-iNET ORION spheroids showed spherical tumors with average diameter around 12–15 mm when sacrificed 6 months after transplantation (Fig. 3h–j).

## Xenograft histology

Confocal imaging of 10 µm-thick cryosections, prepared from xenografts and stained with fluorescently labeled pan-cytokeratin antibody showed clear staining in the area of NET cells but not surrounding stroma. Expression of Synaptophysin was also detected in xenograft cryosections. H&E staining of xenografted 3D-iNET ORION cells showed similar characteristics with H&E staining from original human tumor biopsy (Fig. 3k, l).

## Human Core Exome Panel Analysis

Array data has confirmed that all three analyzed samples had abnormal karyotypes (as expected for a tumors and tumor cell line) and were derived from the same patient (sample identity matching across 51 SNPs). The karyotypes of 3D-iNET ORION (passage no.13) and 3D-iNET ORION (passage no.20) were identical, while minor differences were observed in sample from primary tumor. A small deletion on Chr6p was only observed for the primary tumor, while both 3D-iNET ORION samples had a ~ 75 Mb deletion on Chr3p and duplication on Chr3q. The list of all detected karyotype abnormalities is shown in a Supplementary Fig. 2 and Supplementary Table 2.

## Discussion

The lack of suitable in vitro cell models for human pancreatic neuroendocrine tumors limits the research progress and development of effective therapies for this type of cancer. While some mouse and rat cell lines are available [9, 10], more human in vitro model systems are needed for NET tumor research. Hereby, we describe newly derived neuroendocrine tumor cell line (“3D-iNET ORION”) isolated from liver metastasis of patient suffering pancreatic neuroendocrine tumor and grown under 3D culture conditions. To the best of our knowledge, 3DiNET ORION is the only human liver metastasis pancreatic neuroendocrine tumor cell line grown in 3D and maintained in serum free cell culture media from the time of isolation from patient biopsy.

With the use of optimized protocol, we maintained the cells and spheroids under 3D culture conditions from the time of isolation from tumor biopsy. The rationale for this approach was based on the recent evidence showing that cancer cells cultured in 3D culture more closely resemble in vivo tissue architecture when compared to traditional two-dimensional monolayer culture. In in vivo environment, cells are typically three-dimensionally surrounded by other cells and extracellular matrix in 3D fashion [11, 12]*.* Testing the new drugs and compounds on cells growing in 2D contributes on the fact that almost 90% of tested chemicals do not go through clinical development. The 3D-iNET ORION cell line, developed in our lab provides a novel 3D in vitro tool for drug testing. The 3D-iNET ORION spheroids can be successfully xenografted into nude mice and therefore, also represents an excellent tool for in vivo drug testing.

Using electron microscopy, we also evaluated our cell line for presence of neurosecretory granules. These subcellular structures are considered to be the true hallmarks of neurons and neurosecretory cells [13]. These ultrastructural components of neuroendocrine tumors were detected in high numbers in sections prepared from 3D spheroids, strongly supporting the neuroendocrine origin of the cells.

Antibody array protein chip analysis of transcription factors in 3DiNET ORION cells revealed expression of several intracellular transcription factors involved in neuroendocrine cancer pathobiology—Snail, GATA4, FoxA2, Sox-2, PDX-1 and E-cadherin. Briefly, Snail is a transcription factor that induces epithelial–mesenchymal transition—a process exploited by invasive cancer cells. In study done by *Fendrich *et al*.*, Snail expression was found to be present in 59% of NET tumors and contributed to tumor invasion and metastatic spread [14]. Similarly, a high expression of Snail was found to be associated with the invasion and metastatic spread also by another group [15]. GATA4 is a member of GATA transcription factors family that was found to be implicated in pancreatic cancer. GATA4 contributes to pancreatic carcinogenesis probably by its interaction with Hedgehog and Notch signaling pathways [16]. FoxA2 belongs to subclass A of the Forkhead box containing transcription factor family [16]. Both FoxA1 and FoxA2 are essential for terminal differentiation and maturation of many endoderm-derived cells, including α-cells in the endocrine pancreas. FoxA1 and FoxA2 were found to be expressed in most stages of pancreatic ductal adenocarcinoma progression [17]. FoxA2 (neuroendocrine-specific transcription factor) was shown to promote tumorigenesis in cooperation with HIF1α in neuroendocrine prostate tumors [18]. Recently, FoxA2 was demonstrated to be a sensitive and specific marker for small cell neuroendocrine carcinoma diagnosis [19].

Sox2 (Sex-determining factor, also known as one of the “Yamanaka factors”) is a transcription factor that regulates pluripotency in stem cells. In cancer, Sox2 is associated with “stemness” of cancer cells and prediction of poor outcome. In study performed by Sholl et al*.* [20]*,* Sox-2 was strongly expressed in 23% of low-grade and 72% of high-grade neuroendocrine carcinomas. E-cadherin is a member of large family of genes coding for calcium-dependent cell adhesion molecules. This highly conserved gene plays a major role in malignant cell transformation. Loss of function of E-cadherin tumor suppressor protein was correlated with the increase of invasiveness and metastasis of tumors [21]. This is typically associated with increase of N-cadherin in epithelial-to-mesenchymal transition (EMT).

In conclusion, 3D-iNET ORION represents a novel three-dimensional cell line for human pancreatic NET that can be cultured and propagated in the form of spheroids, thus mimicking in vivo conditions more closely than traditional 2D culture [22].

### Supplementary Information

Below is the link to the electronic supplementary material.

Supplementary Table.1 The list of antibodies used for immunohistochemistry, fluorescent microscopy, flow cytometry and Western blot analysis. Supplementary file1 (TIF 69 KB)

Supplementary file2 (TIF 16351 KB)

Supplementary Fig.1 STR analyses of primary tumor and 3D-iNET ORION cancer cell line. Supplementary file3 (TIF 115 KB)

Supplementary Fig.2 Human Core Exome Array Analysis of 3D-iNET ORION cell line (Passage No.13 and No.20) and original tumor showing abnormal karyotype and sample identity data. Samples were run in quadruplicates. Supplementary file4 (XLSX 15 KB)
