# Supplementary figures and images for: Development of 3D-iNET ORION: a novel, pre-clinical, three-dimensional in vitro cell model for modeling human metastatic neuroendocrine tumor of the pancreas
Source: Hum Cell. 2024 Aug 5;37(5):1593–601. doi: 10.1007/s13577-024-01113-7 (PMC11341600; doi:10.1007/s13577-024-01113-7)

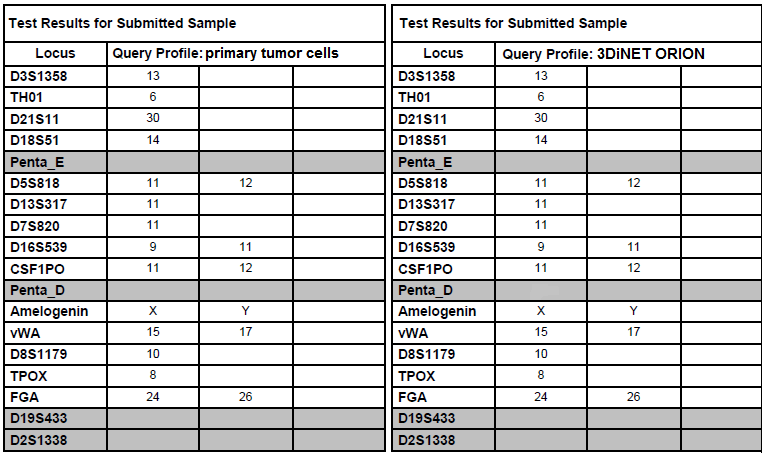

Supplement: Supplementary file 1 — Supplementary Table.1 The list of antibodies used for immunohistochemistry, fluorescent microscopy, flow cytometry and Western blot analysis. Supplementary file1 (TIF 69 KB) [file 13577_2024_1113_MOESM1_ESM.tif]

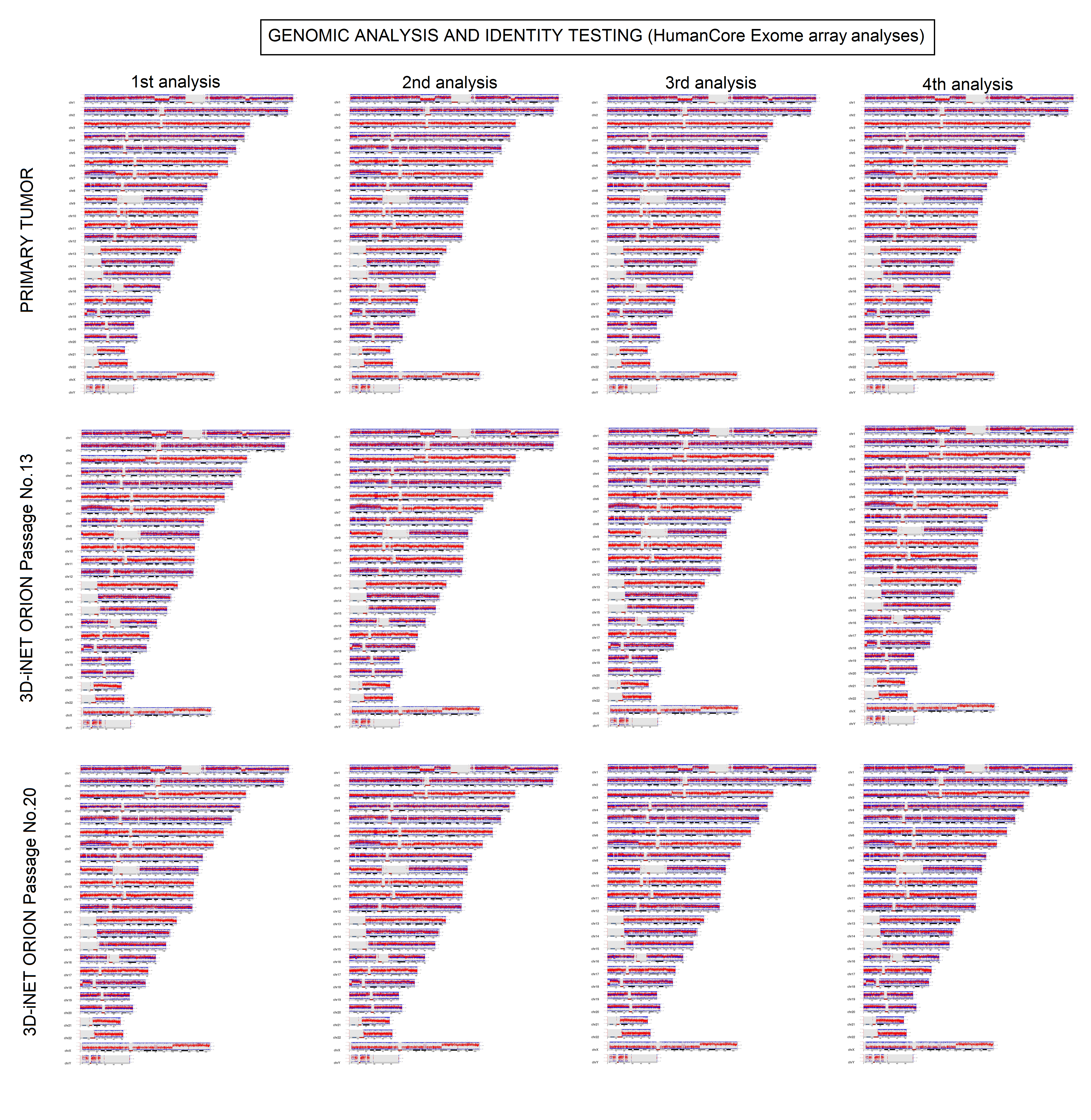

Supplement: Supplementary file 2 — Supplementary file2 (TIF 16351 KB) [file 13577_2024_1113_MOESM2_ESM.tif]

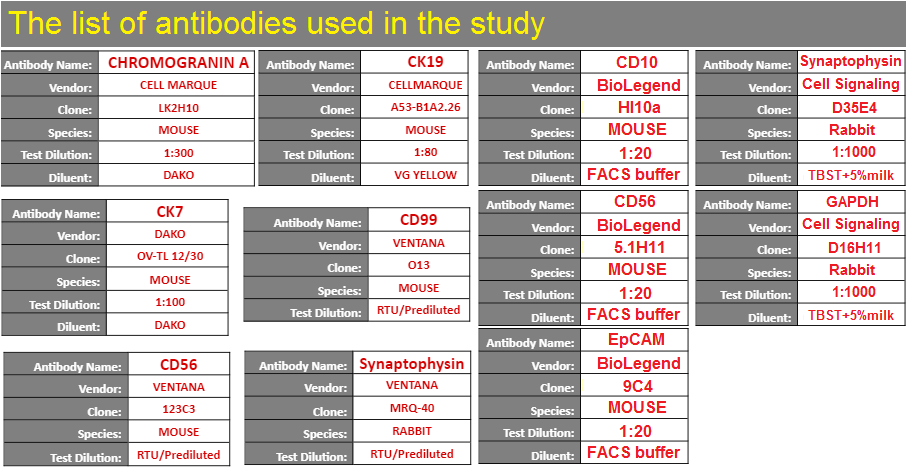

Supplement: Supplementary file 3 — Supplementary Fig.1 STR analyses of primary tumor and 3D-iNET ORION cancer cell line. Supplementary file3 (TIF 115 KB) [file 13577_2024_1113_MOESM3_ESM.tif]
